# High-dose pulsed hyaluronidase for managing nasal skin necrosis following hyaluronic acid treatment in nasolabial folds: A case report

**DOI:** 10.1016/j.jobcr.2024.04.006

**Published:** 2024-04-22

**Authors:** Marcelo Germani, Panmella Alegria, Gabriela Giro, Victor R.M. Munoz-Lora

**Affiliations:** a Department of Periodontology and Implantology, University of Guarulhos, São Paulo, Brazil; b Private Office, São Paulo, Brazil; c Let's HOF Academy, São Paulo, Brazil

**Keywords:** Hyaluronic acid, Ischemia, Necrosis

## Abstract

The growing popularity of soft tissue filler injections has brought attention to the associated risks, particularly vascular complications, and their treatments. This case report focuses on a 34-year-old female who developed nasal skin necrosis following hyaluronic acid (HA) filler injection for nasolabial fold (NLF) enhancement. Despite the careful procedure, complications emerged rapidly, emphasizing the critical importance of prompt diagnosis and intervention. A total of 10,000 turbidity reducing units (TRU) of hyaluronidase (HSE) were administered in a high-dose pulsed manner, alongside hyperbaric oxygen therapy. The patient experienced a gradual but significant improvement over 60 days. This case underscores the need for constant vigilance in aesthetic medicine and the potential consequences of even minute HA amounts, exceeding zero, in causing severe vascular events.

## Graphical abstract

Patient evolution of nasal skin necrosis. A) Twelve days after hyaluronic acid treatment, B) 6 days after hyaluronic Acid treatment and hyaluronidase application and C) 60 days after hyaluronic acid treatment and hyaluronidase application.

**Figure:**
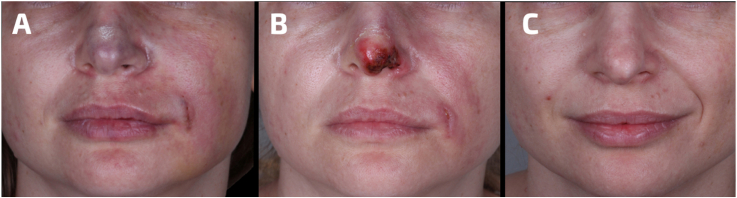

## 1 Introduction

According to the Aesthetic American Society, the number of soft tissue filler injections increased by 42 % between 2020 and 2021, reaching a total of 1,857,339 procedures in the United States in 2021. In the same period, the number of procedures for reversing filler treatments also increased by 57 %, with 23,031 corrective interventions in 2021. The Manufacturer and User Facility Device Experience (MAUDE) database reported that skin necrosis accounted for 3.5 % of all adverse events after fillers injection. Moreover, the nasolabial folds (NLF) and nose were the two more common sites related to skin necrosis, with 20.8 % and 15.6 % respectively. In the same way, vision changes accounted for 1.5 % of all adverse events, with the nose (20.2 %) and NLF (15 %) areas again as the top injection sites.^1^

Adverse effects caused by hyaluronic acid (HA) applications, are commonly treated using hyaluronidase (HSE).^2^ This enzyme breaks down the peptide bonds within long-chain in HA molecules, therefore degrading the product. However, a positive outcome in reversing these adverse events depends on the prompt response of the clinician to diagnose and treat the complication. A consensus opinion in the literature states that 5 turbidity reducing units (TRU) of HSE are needed to decompose 0.1 mL of a filler with 20 mg/mL of HA.^3^ However, when there is a risk of tissue loss (necrosis) or amaurosis (blindness), larger doses of HSE (2000–3000 TRU) at very short intervals (15–20 min) are suggested.^4^^,^^5^

Hence, this article aimed to present a successfully treated case of nasal skin necrosis due to HA filler injections using a high-dose pulsed HSE protocol.

## 2 Case report

A 34-year-old female patient visited a private clinic in São Paulo, Brazil, complaining about deep NLFs and seeking facial rejuvenation procedures. Following an initial assessment and planning, the treatment comprises the use of HA fillers into the NLFs.

To fill the NLFs, a cannula entry point was done using a 22G needle (Terumo, Brazil) at the beginning of each fold. Topical anesthesia (Pliaglis, Galderma, Sweden) was used to reduce the needle-prick pain. Then, 0.5 mL of Restylane Volyme (Galderma, Upsalla, Sweden) was subcutaneously applied using a fanning technique with a 22G cannula (Biometik, Biomethyl, Santa Catarina, Brazil) on each side. The cannula was oriented towards the paranasal space and retro injections were done until 0.5 mL of the product was deposited on each side. Post procedures instructions were passed, and the patient returned home.

Four hours post-application, the patient returned to the clinic complaining of moderate pain and color change in the left paranasal region. A compression test to assess vascular perfusion of the affected area was performed and ischemia of the region was detected. Then, the administration of HSE to degrade the product was decided. For this, 3000 TRU of lyophilized HSE (Next Pharma, Santos, Brazil) were reconstituted using 3 mL of sterile saline solution. All the reconstituted HSE was retro injected into the affected area using a 22G microcannula (Biometik, biomethyl, Santa Catarina, Brazil).

**Fig. 1:**
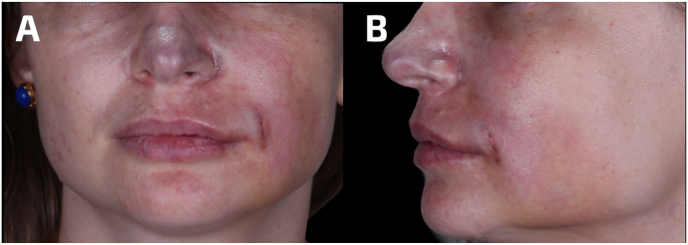
Assessment of the patient 12 h post-treatment in a A) frontal and B) left lateral view. Changes in skin color on the left side of the face and lips can be observed.

Twelve hours later, the patient still experienced severe pain in the left NLF, along with changes in skin color on the face and lips. Additionally, the nasal tip became numb (Fig. 1A and B). After considering the reported signs and symptoms, it was decided to administer HSE again.

This time, high-dose pulsed HSE injections were performed.^2^^,^^5^ For this, 2000 TRU of a different HSE (Biometil, Santa Catarina, Brazil) were diluted into 0.5 mL of sterile saline solution and applied subcutaneously into the affected area using a 22G microcannula. A firm massage was applied to the region after HSE injection. Then, a new compression test was performed to verify the ischemia process. If the ischemia was still present, a new application of the same high-dose pulsed HSE was performed until no sign of ischemia was found anymore. A total of 10000 TRU of HSE were administered (Table 1). Finally, the patient underwent a Doppler ultrasound examination (P5 color Doppler, GE Healthcare, Brazil) to confirm the clearance of the affected region.

**Table 1:** Quantity of interventions and the total amount of hyaluronidase administered to the patient.

Application	HSE Brand	Amount	Dilution	Time interval between applications
First	Next Pharm	3000 U	3 mL diluent	4 h after procedure
Second	Biometil	2000 U	0.5 mL diluent	12 h after procedure
Third	Biometil	2000 U	0.5 mL diluent	20 min after the 2nd application
Fourth	Biometil	2000 U	0.5 mL diluent	20 min after the 3rd application
Fifth	Biometil	2000 U	0.5 mL diluent	20 min after the 4th application
Sixth	Biometil	2000 U	0.5 mL diluent	20 min after the 5th application

**Fig. 2:**
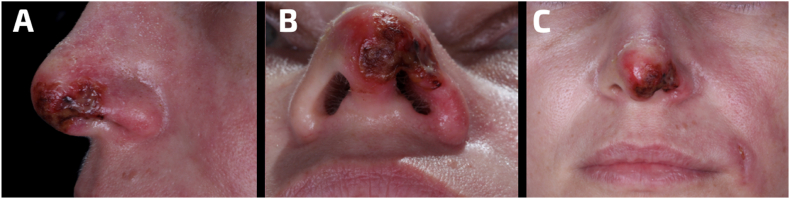
Assessment of the patient's nose from a A) lateral, B) inferior and C) frontal view 6 days post-treatment. The nose exhibited characteristics of advanced scarring.

Nonetheless, 72h after the initial filler injection the patient was still experiencing significant pain on the left side, therefore, Ibuprofen 400 mg every 6 h for 5 days was prescribed. Additionally, the patient started a 60-min hyperbaric oxygen therapy twice a day at 202.6 kPa for one week. Four days post-filler, pustules were detected on the left nasal wing, with a purplish discoloration at the nasal tip. Six days after the initial filler application, the alar region of the nose exhibited characteristics of advanced scarring, so it was recommended a topical application of a hydrogel containing fatty acids and vitamins A and E (Dersani ointment, MegaLab Brazil, Brazil) (Fig. 2A–C).

**Fig. 3:**
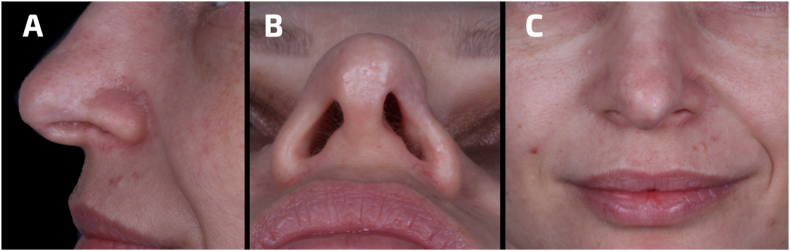
Assessment of the patient's nose from a A) lateral, B) inferior and C) frontal view 60 days post-treatment. The nose exhibited characteristics of recovery.

The patient continued with complementary therapies (hyperbaric oxygen therapy twice a week and topical application of Dersani) for 60 days, finally resulting in a significant improvement of the affected region (Fig. 3A–C).

## 3 Discussion

Numerous reports have documented facial and alar necrosis following HA injections into the NLF and nose.^6^^,^^7^ These complications may occur following intravascular HA injections or arterial compression, and the vascular anatomy of the region plays a critical role. The lateral nasal artery, a branch of the facial artery, provides blood supply to the nasal ala and dorsum. For this reason, the blood supply in the external nose holds significant clinical relevance concerning filler injections in the NLF.^8^ This connection may explain the possible cause of the complication in our report.

Even with good anatomical knowledge and a correct technique, there is a constant risk of vascular events using HA, even within highly skilled and experienced injectors. The natural progression of untreated vascular embolic events involves momentary blanching (starting fast), livedo reticularis (up to several days), blistering (from the third day onward), crusting, necrosis, and eventual healing by secondary intention, a process that may last six weeks or longer.^9^

Some precautions like injecting slowly and the use of low volumes of HA, should be followed to avoid a possible vascular embolic event. However, all clinicians must be prepared to treat possible complications. HSE applications are the main treatment of vascular complications with HA, for this reason, in any office that routinely performs soft tissue augmentation with HA fillers, HSE should be accessible.^4^ Recent reports suggest the necessity of high-dose pulsed HSE injections for successful reversal of vascular events following HA injections.^2^^,^^4^^,^^5^ By this means, we ensure to have sufficient concentration of HSE around the affected tissue. Though, complimentary therapy (*i.e.* hyperbaric oxygen therapy and topical application of a wound healing gel) also plays a critical role in wound healing after addressing the vascular event.

In this report, an initial approach with a conventional HSE treatment proved to be unsuccessful. It is important to recognize that HA products degrade at varying rates, often assessed by in-vitro tests, which do have their limitations.^10^^,^^11^ However, it is worth mentioning that the HA product used in this report has shown satisfactory degradation rates in prior studies. Nevertheless, factors such as the HA volume injected, the specific HSE used, and the actual interaction conditions (which differ from in-vitro tests) could explain the failure of the initial treatment attempt and may affect the degradation time of HA gels.^10^

Further research is necessary to elucidate the interactions between HA and HSE, particularly as new HA products emerge and the usage of HSE increases. It's noteworthy that even minuscule amounts of HA above zero can trigger obstructive or compressive vascular events, potentially resulting in irreversible complications for patients.^12^

## 4 Conclusion

This case report highlights the significant risk of vascular complications, including necrosis, following HA injections in the NLF due to the complex vascular anatomy. It is crucial for clinicians to remain vigilant and well-prepared for swift and precise interventions, as even minuscule amounts of HA can trigger severe vascular events. Notably, high-dose pulsed HSE injections have emerged as a promising and effective treatment to address these complications.

## Source of funding

This case report received no funding.

## Funding

This study received no funding.

## Declaration of competing interest

The authors report no conflict of interests.
